# Random heterogeneous spiking neural network for adversarial defense

**DOI:** 10.1016/j.isci.2025.112660

**Published:** 2025-05-14

**Authors:** Jihang Wang, Dongcheng Zhao, Chengcheng Du, Xiang He, Qian Zhang, Yi Zeng

**Affiliations:** 1 Brain-inspired Cognitive AI Lab, Institute of Automation, Chinese Academy of Sciences, Beijing, China; 2 School of Artificial Intelligence, University of Chinese Academy of Sciences, Beijing, China; 3 School of Future Technology, University of Chinese Academy of Sciences, Beijing, China; 4 State Key Laboratory of Brain Cognition and Brain-inspired Intelligence Technology, Shanghai, China; 5 Beijing Key Laboratory of AI Safety and Superalignment, Beijing, China; 6 Beijing Institute of AI Safety and Governance, Beijing, China; 7 Center for Long-term AI, Beijing, China

**Keywords:** Physics, Computer science, Engineering

## Abstract

Spiking neural networks (SNNs) offer a biologically inspired alternative to artificial neural networks (ANNs) by mimicking neuronal information transmission mechanisms. However, similar to ANNs, SNNs remain susceptible to adversarial examples, raising concerns about their robustness in practical applications. To address this vulnerability, we propose the Random Heterogeneous Spiking Neural Network (RandHet-SNN), inspired by the heterogeneity and stochasticity observed in biological neural systems. This architecture strengthens the network’s defense against adversarial attacks by introducing neuron-level diversity through randomized time decay constants, allowing each neuron to acquire unique temporal properties at every forward pass. We evaluate RandHet-SNN’s performance through extensive experiments with various adversarial attacks. Results indicate that RandHet-SNN significantly enhances network robustness while maintaining minimal impact on clean accuracy. RandHet-SNN displays significant potential for robust, energy-efficient neural computing in adversarial environments.

## Highlights

• Incorporating neuronal heterogeneity and trial-to-trial spiking variability
• Random time constant significantly enhances adversarial robustness
• RandHet-SNN outperforms randomization methods in ANN

## Graphical abstract

**Figure:**
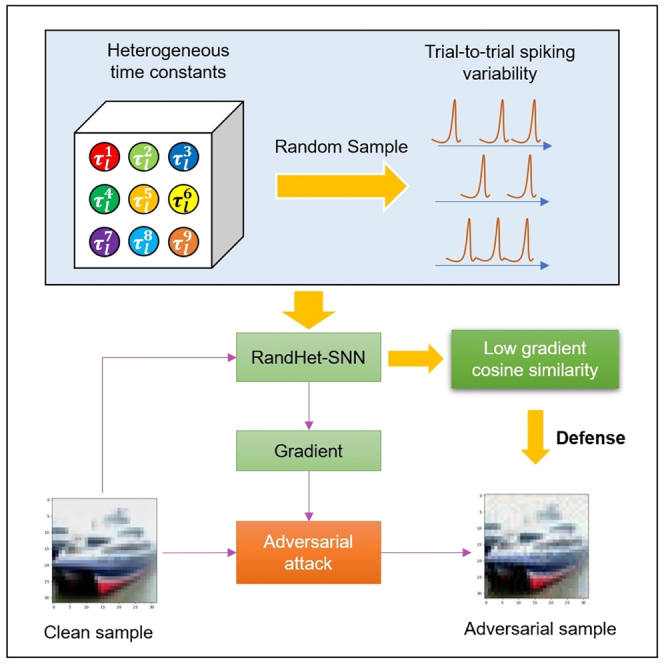

## Introduction

Deep neural networks have demonstrated remarkable success in processing high-dimensional data, enabling significant breakthroughs in areas such as computer vision and natural language processing.^1^^,^^2^ However, concerns regarding the robustness of DNNs, particularly their vulnerability to adversarial attacks, remain a major limitation.^3^^,^^4^ In image classification, for instance, adversarial attacks can introduce subtle perturbations that are nearly imperceptible to human observers yet cause models to misclassify inputs. This vulnerability critically undermines the reliability of DNNs in safety-critical applications, such as autonomous driving,^5^ highlighting a fundamental discrepancy between the perception mechanisms of DNNs and the human visual system.

Incorporating stochastic mechanisms is a promising approach to enhancing adversarial robustness in neural networks.^6^^,^^7^^,^^8^^,^^9^^,^^10^^,^^11^ Xie et al. demonstrated that preserving randomness in input padding and resizing during inference disrupts attack patterns, thereby improving resilience to adversarial perturbations.^6^ A widely adopted approach, randomized smoothing, adds Gaussian noise to the network inputs offering certified $L_{2}$ robustness against adversarial attacks.^11^ Dong et al. observed that adversarial attacks exhibit limited transferability across networks with different normalization methods, which led to the development of the Random Normalization Aggregation (RNA) approach. This method forces attackers to adopt black-box strategies, thereby strengthening defensive capabilities.^8^ Random Projection Filters (RPF) apply random projections within convolutional layers, enabling random sampling during both training and inference to boost robustness by increasing stochasticity in the network.^9^ Ma et al. introduced Constrained Trainable Random Weights (CTRW) to increase gradient variance during inference, which improved adversarial defense but also led to a trade-off in clean accuracy.^10^

Traditional artificial neural networks (ANNs) rely on floating-point values to represent neuron firing rates, whereas spiking neural networks (SNNs) model neural activity through discrete temporal spikes, closely mimicking biological neural behavior. This spiking mechanism allows SNNs to achieve higher energy efficiency on neuromorphic hardware, making them more suitable for power-constrained applications.^12^^,^^13^ Specifically, neurons in SNNs have binary activation states (0 or 1), allowing matrix multiplications in forward propagation to be executed with additions, thereby reducing energy consumption. Despite their advantages in energy efficiency and biological plausibility, SNNs are similarly vulnerable to adversarial perturbations.^14^^,^^15^ Various strategies have been explored to improve adversarial robustness in SNNs. Adversarial defenses in SNNs draw inspiration from methods used in ANNs while also leveraging the distinct characteristics of SNNs, including adversarial training,^16^^,^^17^ certified training,^18^ regularization-based methods^19^^,^^20^ and random smoothing method.^21^ Ding et al. examined biological principles related to stochastic gating mechanisms in synapses and introduced randomness into the input currents of neurons. This stochastic modulation of input currents was found to enhance adversarial robustness in SNNs by incorporating biological insights into synaptic variability.^22^

Notably, neural activity in the visual cortex of the biological brain exhibits substantial randomness,^23^ suggesting a potential link between stochastic information processing and robustness. In response to identical stimuli, neuronal activity in the nervous system varies due to intrinsic randomness, enabling the system to filter out environmental noise and focus on essential information.^24^

Additionally, unlike most ANNs and SNNs, which typically employ standardized activation functions or membrane potential dynamics, biological neural networks exhibit marked heterogeneity in their electrophysiological properties. The nervous system comprises diverse neuron types, and this variability contributes to system-level redundancy and fault tolerance. When certain neuron types are damaged, other neurons can partially compensate for their functions, enabling the system to maintain functionality.^25^ Research suggests that this diversity in neuronal parameters enhances network robustness.^26^^,^^27^

Previous works have primarily introduced random parameters in the weights, biases, or normalization layers of ANNs, rather than randomizing the parameters within the activation function, which does not highlight the heterogeneity of neurons in the network.^6^^,^^7^^,^^8^^,^^9^^,^^10^ To overcome these limitations, this paper presents the Random Heterogeneous Spiking Neural Network (RandHet-SNN), an architecture that incorporates stochasticity and heterogeneity in neuronal responses by randomizing neuron time constants within SNNs. The random time constants can introduce neuronal heterogeneity into the network, which is a potential factor for enhancing the robustness of the network’s information processing. We evaluate RandHet-SNN on CIFAR-10 and CIFAR-100 datasets^28^ under a range of adversarial attack methods. Experimental results demonstrate that RandHet-SNN provides substantial improvements in both adversarial robustness and clean accuracy, effectively complementing adversarial training techniques. Compared to previous works based on random parameters, RandHet-SNN shows better clean accuracy and robust accuracy under attacks based on Expectation Over Transformation (EOT). These findings suggest that incorporating stochastic and heterogeneous features within SNNs enhances their defensive capabilities against adversarial attacks while also improving baseline performance.

## Results

### Random heterogeneous spiking neural network

**Figure 1:**
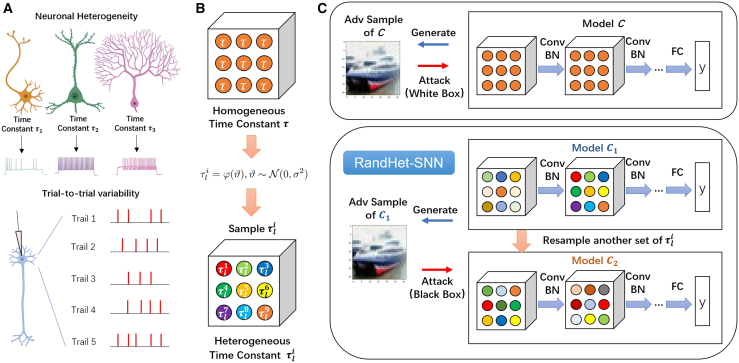
A conceptual diagram illustrating the biological inspiration behind the RandHet-SNN, its overall architecture, and the adversarial defensive mechanism (A) **Top**: Biological neurons exhibit diverse electrophysiological properties. For the LIF model, neuronal heterogeneity can be achieved through different neuron time constants τ. **Bottom**: The spiking responses to the same input vary across different trials. This trial-to-trial variability is induced by randomness inherent in the nervous system. (B) In a vanilla SNN, all neurons have the same time constant, whereas in RandHet-SNN, each neuron independently samples its time constant during each forward pass, resulting in a heterogeneous network after sampling. (C) Diagram of the RandHet-SNN. **Top**: In vanilla SNNs, adversaries can generate white-box attacks on the model using network gradients. **Bottom**: RandHet-SNN samples random time constants $\tau_{l}^{i}$ to generate a specific set of network parameters, represented by model $C_{1}$. The attacker uses the gradients of $C_{1}$ to create adversarial examples. However, when these adversarial examples are input into RandHet-SNN, the time constants $\tau_{l}^{i}$ are resampled, resulting in a new network denoted as model $C_{2}$. Consequently, the adversarial perturbations generated based on $C_{1}$ may not remain effective against $C_{2}$.

Randomness in information processing and neuronal heterogeneity are foundational characteristics of biological neural networks. Different types of neurons often exhibit distinct electrophysiological properties. Due to inherent randomness in the nervous system, neurons may display varied spiking responses to identical inputs, as shown in Figure 1A. In SNNs with leaky integrate-and-fire (LIF) neurons, the time constant is a crucial parameter that regulates each neuron’s membrane potential dynamics. Therefore, neuronal heterogeneity can be achieved through different neuron time constants τ.^26^ Building on this, we propose the RandHet-SNN, which incorporates both randomness and heterogeneity by randomizing neurons’ time constants. This design enables RandHet-SNN to emulate biological neural networks more effectively, introducing diversity in neuronal parameters and randomness in information processing.

In RandHet-SNN, each neuron within every network layer is assigned an independent random variable as its time constant. During forward propagation, these time constants are sampled from a predefined distribution, allowing neurons across the network to exhibit both heterogeneity and randomness.

We define each neuron’s time constant as an independent random variable. In this paper, two sampling methods for the time constants are utilized. The first method involves independently sampling the time constants at each time step, while the second method samples the time constants at the beginning of each forward pass maintaining them as constant for all subsequent time steps.

RandHet-SNN uses the first sampling method by default and we use RandHet-${\text{SNN}}^{*}$ to indicate that the model employs the second sampling method. Each neuron samples independently during each forward propagation, as illustrated in Figure 1B. This independent sampling mechanism allows each neuron to exhibit distinct dynamic characteristics during different forward propagation processes, thereby enhancing the network’s randomness and heterogeneity.

**Figure 2:**
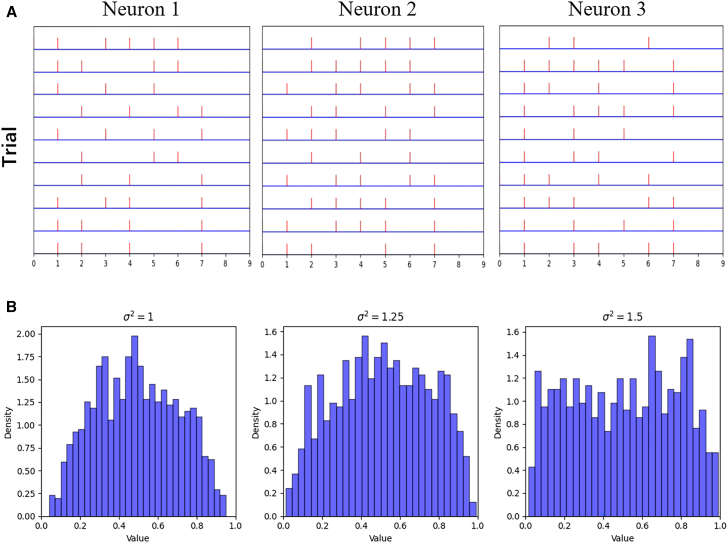
The trial-to-trial variability and time constants distribution in RandHet-SNN (A) The spike trains of neurons across different trials under the same input. The figure shows that neuron responses with random time constants exhibit trial-to-trial variability. (B) Distribution of time constants for different variances σ. Each subfigure shows τ values (*x* axis) versus their probability density (*y* axis), with density calculated as $\left(n_{i}\times \Delta \tau \right)$, where $n_{i}$ is the sample count in the i-th bin and Δτ is the bin width.

The Figure 2A shows the spike sequences of three neurons in RandHet-SNN under the same input across different trials. As seen in the figure, the random LIF neurons in RandHet-SNN exhibit strong trial-to-trial variability, which is consistent with the response characteristics of biological neurons.^23^^,^^29^

We further examine the distribution of time constants under varying values of variance, as shown in Figure 2B. When the variance is set to 1.5, the distribution of time constants approximates a uniform distribution over the interval (0,1). As variance decreases, time constant values concentrate increasingly around 0.5.

The stochastic mechanism in RandHet-SNN disrupts an attacker’s ability to determine the network’s deterministic parameters, effectively transforming a white-box attack into a black-box scenario, as illustrated in Figure 1C.

### Performance

#### Experimental settings

In this study, we evaluate the adversarial robustness of RandHet-SNN on the CIFAR-10 and CIFAR-100 datasets. The SNNs are implemented using the SEW-ResNet19 architecture,^30^ where neurons are modeled as LIF neurons with randomized time constants, a variance of $\sigma^{2}=1$, a threshold of $V_{th}=0.5$, and a time step of T=8. RandHet-SNN is trained for 100 epochs on each dataset.

To comprehensively evaluate the performance of RandHet-SNN when combined with different adversarial training methods, we use three adversarial training techniques for SNNs: standard adversarial training (AT), regularized adversarial training (RAT)^16^ and sparsity regularization(SR).^20^ Among these, the RAT and SR methods are well-established and effective techniques for training robust SNNs. For AT, we introduce $L_{2}$ regularization to enhance the network’s robustness, with a regularization coefficient of $1\times 10^{-4}$. For RAT and SR, the regularization coefficient is set to 0.004 and 0.001, respectively. Adversarial training samples are generated using the RFGSM method with a perturbation magnitude of ε=4/255, based on the $L_{\infty }$-norm.

#### Adversarial attack settings

We evaluate RandHet-SNN’s robustness under multiple adversarial attack methods, including FGSM, PGD, AutoPGD (APGD), MIFGSM,^31^ and AutoAttack,^32^ with a perturbation magnitude of ε=8/255. ${\text{PGD}}_{k}$ and ${\text{APGD}}_{k}$ are used to denote PGD and APGD attacks with k iterations, such as ${\text{PGD}}_{10}$, where the step size per iteration is α=2ε/k. For MIFGSM, the step size is 2/255 with a decay factor of 0.1 across 5 iterations.

To rigorously assess RandHet-SNN’s adversarial robustness, we address gradient obfuscation effects when generating white-box adversarial samples. In each attack iteration, we apply the EOT method^33^ to obtain more accurate gradient estimates. We use ${\text{PGD}}_{n}^{EOTm}$ to represent the PGD attack with an iteration step size of n and EOT iteration count of m. This approach allows for a more precise evaluation of the model’s robustness against adversarial inputs.

#### Robustness against adversarial attacks

To evaluate adversarial robustness across multiple attack methods, we use the minimum robust accuracy (MRA) as the primary metric, as it provides the most rigorous assessment of the model’s resilience. The MRA captures the model’s performance under the strongest adversarial attacks, effectively reflecting its robustness in worst-case scenarios. This metric is thus crucial for a comprehensive evaluation of the model’s robustness across various adversarial settings. Table 1 summarizes the robust accuracy of RandHet-SNN and RandHet-${\text{SNN}}^{*}$ trained with AT, RAT and SR, alongside that of vanilla SNNs with the same architecture, under different adversarial attacks. The numbers higher than the baseline are indicated in bold in the table.

**Table 1:** Comparison of CIFAR-10 and CIFAR-100 performance across different adversarial attacks

Attack	Clean	FGSM	PGD_10_	APGD_10_	MIFGSM	AutoAttack	MRA^a^
**CIFAR-10**							

SNN+AT	86.38	49.48	41.10	33.31	45.24	29.29	29.29
RandHet-SNN+AT	**87.36**	**50.13**	**41.79**	**47.60**	45.12	**48.59**	**41.79**
RandHet- ${\text{SNN}}^{*}$ +AT	**87.29**	**52.58**	**44.33**	**50.65**	**46.73**	**50.34**	**44.33**
SNN+RAT	89.55	52.21	42.09	34.49	46.76	30.36	30.36
RandHet-SNN+RAT	**90.25**	**53.53**	**44.86**	**47.20**	**47.46**	**47.76**	**44.86**
RandHet- ${\text{SNN}}^{*}$ +RAT	**89.88**	**54.74**	**46.04**	**49.80**	**48.12**	**51.57**	**46.04**
SNN+SR	87.13	50.41	43.35	36.08	46.42	32.96	32.96
RandHet-SNN+SR	**88.11**	**50.98**	**44.19**	**47.54**	**46.76**	**47.45**	**44.19**
RandHet- ${\text{SNN}}^{*}$ +SR	**88.19**	**53.82**	**47.36**	**51.26**	**49.37**	**51.61**	**47.36**

**CIFAR-100**							

SNN+AT	63.13	25.04	20.08	15.99	22.59	12.79	12.79
RandHet-SNN+AT	**64.10**	**25.27**	19.90	**25.64**	22.09	**25.31**	**19.90**
RandHet- ${\text{SNN}}^{*}$ +AT	**63.58**	**25.79**	**21.07**	**28.34**	22.19	**27.22**	**21.07**
SNN+RAT	68.12	30.33	23.89	19.17	26.76	15.14	15.14
RandHet-SNN+RAT	**68.45**	**31.84**	**25.56**	**28.88**	**27.45**	**27.12**	**25.56**
RandHet- ${\text{SNN}}^{*}$ +RAT	67.96	**31.98**	**26.19**	**30.27**	**27.03**	**30.13**	**26.19**
SNN+SR	63.60	25.77	21.47	17.04	23.71	13.82	13.82
RandHet-SNN+SR	**64.46**	**26.33**	**21.88**	**25.46**	23.09	**25.31**	**21.88**
RandHet- ${\text{SNN}}^{*}$ +SR	**65.09**	**27.93**	**23.29**	**28.73**	**24.89**	**28.44**	**23.29**

The data in the table represents the accuracy under the corresponding attacks.

a Minimum Robust Accuracy (MRA).

As shown in Table 1, training vanilla SNNs with AT, RAT and SR methods yields limited adversarial robustness against APGD and AutoAttack. In contrast, RandHet-SNNs not only improve model accuracy but also significantly enhance robust accuracy under these attacks. Specifically, RandHet-SNN with the RAT method and RandHet-${\text{SNN}}^{*}$ with the SR method achieve consistently higher robust accuracy across all attacks on both CIFAR-10 and CIFAR-100 datasets, with particularly notable improvements against APGD and AutoAttack. For the AT and SR method, although RandHet-SNNs show a slight decrease in robust accuracy on PGD and MIFGSM, the MRA still demonstrates a marked improvement in adversarial robustness compared to vanilla SNNs. These results demonstrate that both RandHet-SNN and RandHet-SNN∗ can be effectively integrated with various adversarial training methods to enhance the model’s robustness against a wide range of adversarial attacks. Models trained with RAT and SR methods exhibit similar performance, and outperform those trained with AT.

Since the RAT method requires fewer computational resources and less time than the SR method, we use the RAT approach by default for training the model in the subsequent analysis to further evaluate the performance of the RandHet-SNN. We compare the robust accuracy of RandHet-SNN, RandHet-${\text{SNN}}^{*}$, and vanilla SNNs across varying iteration step sizes for PGD and APGD attacks, as well as different EOT step sizes. Varying the PGD and APGD step sizes allows for assessing the model’s resilience under increasingly strong attacks, while adjusting the EOT step sizes helps mitigate the effects of gradient obfuscation in stochastic models.

**Figure 3:**
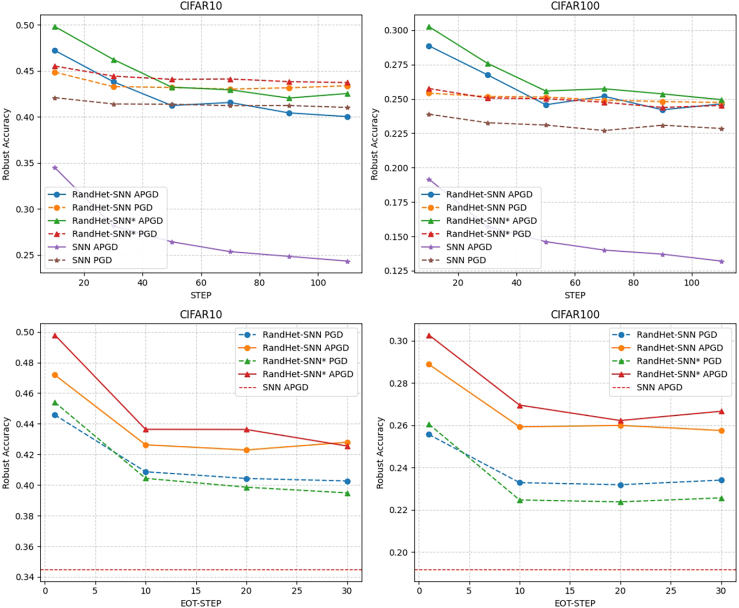
Robust accuracy of RandHet-SNN, RandHet-${\text{SNN}}^{*}$ and vanilla SNNs under different attack settings Robust accuracy of RandHet-SNN, RandHet-${\text{SNN}}^{*}$ and vanilla SNNs under different PGD, APGD iteration step sizes, and EOT step sizes. Robust accuracy refers to a model’s classification accuracy on adversarial examples generated by corresponding adversarial attacks. Dashed lines indicate the robust accuracy under PGD attack, while solid lines represent that under APGD attack. The robust accuracy curves for RandHet-SNN are marked with “·”, RandHet-SNN∗ uses “▲” markers, and SNN is marked with “⋆”.

In Figure 3, the first row illustrates robust accuracy on CIFAR-10 and CIFAR-100 under PGD and APGD attacks with different iteration step sizes. Dashed lines indicate the robust accuracy under PGD attack, while solid lines represent that under APGD attack. As the number of attack iterations increases, the robust accuracy decreases and stabilizes after the step size exceeds 50. Under both PGD and APGD attacks, RandHet-SNN and RandHet-${\text{SNN}}^{*}$ demonstrate higher robust accuracy at each iteration step size compared to vanilla SNNs, with a more substantial improvement under APGD attacks.

The second row shows the robust accuracy of both datasets across varying EOT step sizes. Since vanilla SNNs lack stochastic components, EOT testing is unnecessary for them. In the figure, the horizontal red dashed line represents the robust accuracy of the vanilla SNN under the ${\text{APGD}}_{10}$ attack. Notably, when the EOT step size exceeds 10, the robust accuracy of RandHet-SNNs stabilizes and remains consistently higher than that of vanilla SNNs. RandHet-${\text{SNN}}^{*}$ is more affected by the EOT attack, with its robust accuracy slightly lower than that of RandHet-SNN under the PGD attack with large EOT step size.

**Figure 4:**
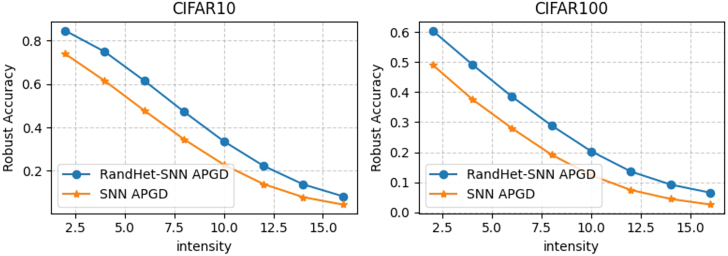
Robust accuracy of RandHet-SNN and vanilla SNN under varying levels of perturbation intensity ε The APGD robust accuracy curves with respect to ε on CIFAR-10 and CIFAR-100. The *x* axis represents the adversarial perturbation size (ϵ) in the pixel space, displayed in the 0–255 scale. RandHet-SNN are marked with “·” and SNN are marked with “⋆”.

Therefore, we conclude that the adversarial robustness of the two models is similar, with RandHet-SNN exhibiting greater robustness than RandHet-SNN∗ when trained with the RAT method. Consequently, we select RandHet-SNN for further analysis of its adversarial robustness under varying levels of perturbation intensity ε. As shown in the Figure 4, RandHet-SNN exhibits higher robust accuracy against the ${\text{APGD}}_{10}$ attack at various perturbation levels compared to the vanilla SNN.

To validate the generalization capability of the RandHet-SNN, we evaluate its performance on the more challenging TinyImageNet dataset.^34^ The results, shown in Table 2, indicate that both the MRA and clean accuracy of the RandHet-SNN outperform those of vanilla SNNs on this dataset, further demonstrating the effectiveness of our approach.

**Table 2:** Comparison of the performance and adversarial robustness of RandHet-SNN and SNN on the TinyImageNet dataset

Attack	Clean	FGSM	MIFGSM	${\text{PGD}}_{50}^{EOT10}$	${\text{APGD}}_{50}^{EOT10}$	MRA
SNN	41.06	24.44	19.32	16.39	7.94	7.94
RandHet-SNN	**50.17**	28.09	21.33	11.17	16.99	**11.17**

### Comparison with randomization methods in ANN

We compare the RandHet-SNN trained with the RAT method to comparable randomization techniques in ANNs (CTRW, RPF, and RNA) on CIFAR-10. All these methods require the generation of adversarial samples for training. To ensure a fair comparison, we control the adversarial sample generation process during training to be consistent across all four randomization methods. The results are shown in the Table 3. In the table, the **Drop** column represents the difference in robust accuracy between ${\text{PGD}}_{20}$ and ${\text{PGD}}_{20}^{EOT10}$. We find that the robustness accuracy of randomization methods in ANNs decreases significantly when subjected to EOT attacks, while the performance drop for the RandHet-SNN remains relatively small. Additionally, RandHet-SNN achieves both higher clean accuracy and higher robust accuracy under the ${\text{PGD}}_{20}^{EOT10}$ attack compared to other ANN methods. This suggests that our RandHet-SNN is a more reliable randomization method.

**Table 3:** Comparison of CIFAR-10 performance across different randomization methods

Attack	Clean	${\text{PGD}}_{20}$	${\text{PGD}}_{20}^{EOT10}$	Drop^a^
CTRW	84.37	**65.53**	27.31	38.22
RNA	89.45	42.72	26.38	16.34
RPF	90.01	40.19	34.62	5.57
RandHet-SNN	**90.12**	43.50	**40.15**	**3.35**

a Drop column represents the difference in robust accuracy between ${\text{PGD}}_{20}$ and ${\text{PGD}}_{20}^{EOT10}$

### Sensitivity to variance

The primary enhancement of RandHet-SNNs over vanilla SNNs arises from the randomization of time constants in the LIF neurons. In the experiments above, we consistently set the variance $\sigma^{2}=1$ for all layers of the network. We further analyze the adversarial robustness of networks with the random time constant mechanism introduced at different layers. In the SEWResNet19 structure we use, the network undergoes three down-sampling stages, dividing the network into three segments. We introduce the random time constant mechanism into each of these three segments separately, with $\sigma^{2}=1$, while the other parts of the network still use LIF neurons with deterministic time constants. We refer to the network with the random time constant mechanism introduced only in the i-th segment as RandHet-${\text{SNN}}_{i}$, and train it by RAT. The results are shown in the Table 4 below.

**Table 4:** The performance of models with the random time constant mechanism introduced at different layers

Attack	Clean	FGSM	MIFGSM	${\text{PGD}}_{50}^{EOT10}$	${\text{APGD}}_{50}^{EOT10}$	MRA
SNN	89.55	52.33	46.76	41.13	26.44	26.44
RandHet-${\text{SNN}}_{1}$	89.68	52.80	47.01	40.38	38.61	38.61
RandHet-${\text{SNN}}_{2}$	88.55	49.97	44.80	38.95	36.55	36.55
RandHet-${\text{SNN}}_{3}$	88.95	51.56	45.86	39.91	31.47	31.47
RandHet-SNN	**90.16**	53.85	47.53	40.40	38.68	**38.68**

As shown in the table, introducing random time constants in the shallow layers of the network results in the most significant improvement in the model’s adversarial robustness compared to vanilla SNN. This highlights the importance of initial data processing for the model’s adversarial robustness. After randomizing all layers of the RandHet-SNN, although the model’s MRA does not show significant improvement, its clean accuracy is further enhanced.

Table 4 demonstrates superior performance when the random mechanism is applied across all layers, so we conducted additional experiments to analyze how the magnitude of random variance affects network performance under this configuration. We train RandHet-SNN with various variances using the RAT method and evaluated their robustness against ${\text{APGD}}_{50}^{EOT10}$ attacks. The results are presented in Table 5.

**Table 5:** Performance comparison on CIFAR-10 and CIFAR-100 across different variance settings under clean and ${\text{APGD}}_{50}^{EOT10}$ attack conditions

Dataset	CIFAR-10		CIFAR-100	
Attack	Clean	${\text{APGD}}_{50}^{EOT10}$	Clean	${\text{APGD}}_{50}^{EOT10}$
$\sigma^{2}=1.0$	90.25	38.70	68.41	23.64
$\sigma^{2}=1.25$	90.40	39.22	67.86	23.54
$\sigma^{2}=1.5$	90.33	38.91	67.58	24.71

The results indicate that RandHet-SNN is relatively insensitive to variations in the time constant’s variance. On CIFAR-100, while the network’s clean accuracy shows a slight decline as variance increases, robust accuracy demonstrates a marginal improvement. These findings suggest that the RandHet-SNN maintains stable performance across varying degrees of $\sigma^{2}$ settings, thereby enhancing its adaptability and robustness.

### Gradient cosine similarity analysis

**Figure 5:**
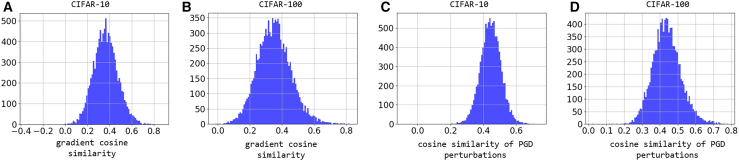
Cosine similarity analysis of gradients and adversarial perturbations (A and B) Distribution of the cosine similarity of gradients between models under different time constants sampling on CIFAR-10 and CIFAR-100. (C and D) Distribution of the cosine similarity of adversarial perturbations between models under different time constants sampling on CIFAR-10 and CIFAR-100. In each figure, the *x* axis represents the range of gradient cosine similarity values, while the *y* axis shows the count of samples falling within each corresponding interval.

The gradients of RandHet-SNN for the same input will vary due to the randomness in the model. Different time constant sampling results produce different gradients for the same input, and we analyze the cosine similarity of these gradient differences. We train RandHet-SNN on CIFAR-10 and CIFAR-100 by RAT and visualize the gradient cosine similarity distributions in Figures 5A and 5B. As shown, the cosine similarity of the gradients primarily clusters around 0.3, indicating that the transferability of adversarial attacks between models with different time constant samplings is relatively low.

Further, let $\delta_{2}$ represent the adversarial perturbation for model $C_{2}$. We evaluate the difference between $\delta_{1}$ and $\delta_{2}$ by measuring their cosine similarity $\cos\left\langle \delta_{1},\delta_{2}\right\rangle$. We generate adversarial perturbations by PGD, and the results are shown in Figures 5C and 5D.

The figure shows that the cosine similarity of adversarial perturbations predominantly falls between 0.4 and 0.5, indicating that the stochastic mechanisms in RandHet-SNN introduce significant variability in the adversarial examples generated by models $C_{1}$ and $C_{2}$. This variability effectively strengthens RandHet-SNN’s adversarial robustness, as attacks designed for one network instance are unlikely to transfer successfully to another due to inherent randomness in neuron parameters.

Moreover, for the RandHet-SNN trained with $\sigma^{2}=1.0$ using RAT on CIFAR-10, we fix all other parameters and vary only the variance of the RandHet-SNN. We then analyze the influence of different random variances on the average gradient cosine similarity (AGCS). As shown in Table 6, larger random variance reduces the AGCS of the RandHet-SNN, while the model’s clean accuracy decreases due to increased randomness. However, the robust accuracy of the model under the ${\text{APGD}}_{50}^{EOT10}$ attack increases as the AGCS decreases. This further demonstrates that the gradient differences induced by the randomness in the RandHet-SNN enhance the model’s adversarial robustness, while also leading to a trade-off between robustness and accuracy.

**Table 6:** The influence of random variance on RandHet-SNN’s Average Gradient Cosine Similarity (AGCS), clean accuracy, and robust accuracy

Variance	AGCS	Clean Acc	${\text{APGD}}_{50}^{EOT10}$ Acc
$\sigma^{2}=0.75$	0.4191	90.58	27.66
$\sigma^{2}=1.0$	0.3659	90.16	38.70
$\sigma^{2}=1.25$	0.3099	88.12	38.74
$\sigma^{2}=1.5$	0.2563	84.59	40.53

### Ablation study

We conduct further ablation experiments to test the adversarial robustness of SNNs when only neuronal heterogeneity is present. Following Xu et al.,^35^ we set the time constants of the neurons in the SNN as trainable parameters, allowing different neurons to learn heterogeneous time constants through training. We refer to this as heterogeneous SNN (Het-SNN). Furthermore, we compare the adversarial robustness of SNNs, Het-SNN, and RandHet-SNN trained with RAT, and the results are shown in Table 7.

**Table 7:** Comparison of the performance of SNN, Het-SNN, and RandHet-SNN under different adversarial attacks

Attack	Clean	FGSM	MIFGSM	${\text{PGD}}_{50}^{EOT10}$	${\text{APGD}}_{50}^{EOT10}$	MRA
**CIFAR-10**						

SNN	89.55	52.33	46.76	41.13	26.44	26.44
Het-SNN	89.38	52.38	46.81	41.34	26.51	26.51
RandHet-SNN	**90.16**	53.85	47.53	40.40	38.68	**38.68**

**CIFAR-100**						

SNN	68.12	30.44	26.76	23.29	14.61	14.61
Het-SNN	67.36	30.13	26.35	22.86	14.60	14.60
RandHet-SNN	**68.36**	31.68	27.67	23.28	23.64	**23.28**

As shown in the table, deterministic heterogeneous neuronal time constants do not improve the performance of the SNN. Therefore, we conclude that the key to the RandHet-SNN’s enhanced adversarial robustness lies in the uncertainty introduced by the random sampling of time constants.

### Computational efficiency analysis

RandHet-SNN requires independent sampling of the time constants for all neurons in the network, which consumes more computational resources compared to a regular SNN. For a network with N neurons and a time step of T, a single forward pass of RandHet-SNN requires NT samples and additional memory to store the time constants for N neurons. We analyze the extra time and memory consumption of RandHet-SNN during training and testing on the CIFAR-10 dataset, as shown in Table 8. In the Table 8, for the training phase, “**Time**” refers to the average time required to train one epoch, while for the testing phase, “**Time**” refers to the time required to complete testing on the test set. “**Memory**” indicates the memory usage during training or testing. All experiments are conducted on NVIDIA A100-PCIE-40GB GPU.

**Table 8:** The additional time cost and memory usage required by RandHet-SNN compared to SNN during training and testing

	SNN	RandHet-SNN	Additional consumption
**Time**			

Train	464.12s	501.18s	7.99%
Test	12.0s	15.0s	25.0%

**Memory**			

Train	17786MB	23959MB	34.7%
Test	1719MB	2125MB	23.6%

For the training phase, “Time” refers to the average time required to train one epoch, while for the testing phase, “Time” refers to the time required to complete testing on the test set. “Memory” indicates the memory usage during training or testing.

As shown in the Table 8, the training time increases by 7.99%, while the inference time increases by 25.0% compared to vanilla SNNs. In terms of memory usage, RandHet-SNN requires 34.7% more memory during training, while the memory overhead during inference is only 23.6% higher than that of standard SNNs. Therefore, we conclude that the additional computational overhead of the RandHet-SNN in practical applications remains within an acceptable range. RandHet-SNN achieves a relatively balanced trade-off between robustness and computational overhead.

The additional computation introduced by RandHet-SNN is limited to independently sampling the time constants for each neuron and does not involve any additional matrix multiplication operations. Therefore, it does not disrupt the original multiplication-free nature of SNNs and can be applied to neuromorphic hardware just like SNNs.

Additionally, we analyzed the sensitivity of the performance of RandHet-SNN and its computational overhead to the network time step. As shown in the Table 9, while the clean accuracy of RandHet-SNN decreases with shorter time steps, its adversarial robustness improves correspondingly. Overall, reducing the time step has a minor impact on RandHet-SNN’s performance but effectively lowers the computational cost during inference. Therefore, RandHet-SNN’s computational overhead can be optimized by appropriately decreasing the time step.

**Table 9:** Comparison of CIFAR-10 performance and computational cost across different time step

Attack	Clean	${\text{PGD}}_{50}^{EOT10}$	${\text{APGD}}_{50}^{EOT10}$	MRA	Time	Memory
T = 4	88.74	40.33	40.13	40.13	10.0s	1570MB
T = 6	89.22	39.40	38.75	38.75	13.0s	1852MB
T = 8	90.16	40.40	38.68	38.68	15.0s	2125MB

## Discussion

This work presents the RandHet-SNN, an approach that leverages stochastic variability in neuron time constants to enhance the adversarial robustness of SNNs. By introducing randomized time constants within LIF neurons, RandHet-SNN effectively emulates the intrinsic stochasticity and heterogeneity found in biological neural systems, resulting in notable improvements in both clean and robust accuracy under various adversarial settings. Extensive evaluations on CIFAR-10 and CIFAR-100 demonstrate that RandHet-SNN consistently outperforms vanilla SNNs, particularly under advanced adversarial attacks such as APGD and AutoAttack. The model’s low sensitivity to changes in variance further validates its stability across hyperparameter variations, emphasizing its resilience and adaptability.

RandHet-SNN also transforms white-box attacks into black-box scenarios, significantly reducing vulnerability to gradient-based attacks. RandHet-SNN demonstrates a marked increase in robust accuracy in conjunction with different adversarial training techniques, underscoring its effectiveness in adversarial defense. These findings suggest that RandHet-SNN holds significant potential for deployment in safety-critical applications, where robustness against adversarial threats is essential. Future work could explore applying similar stochastic mechanisms to other neural architectures and investigating optimal configurations of time constant distributions to further strengthen robustness.

To reveal the potential vulnerabilities of RandHet-SNN, we analyzed adversarial examples that successfully bypassed its defense mechanism. Our findings indicate that these adversarial samples exhibit strong transferability across models with different time constant distributions. Consequently, the randomized time constant mechanism fails to provide effective protection against such examples. To further enhance the adversarial robustness of RandHet-SNN, future work could integrate ensemble-based adversarial defense strategies^36^ to mitigate cross-model transferability of adversarial examples.

### Limitations of the study

In this study, the variance σ is set as a fixed hyperparameter, and it remains the same across all layers in the experiments without varying degrees of randomness assigned to different layers within the network. Although experimental results indicate that RandHet-SNN is relatively insensitive to this hyperparameter, this approach prevents the variance from adapting according to the network structure or depth, limiting RandHet-SNN’s ability to achieve optimal performance across a broader range of network architectures.

We attempted to set σ as a trainable parameter for each layer, optimizing it via the backpropagation algorithm. However, we find that variance optimization based on the backpropagation algorithm causes the variance in each layer to approach 0, which significantly reduces the model’s randomness and weakens the adversarial robustness of RandHet-SNN. This issue is also mentioned in the work of Ma et al.,^10^ who pointed out that backpropagation tends to drive the learnable variance toward 0, making it unsuitable for adaptively learning the appropriate random variance. One possible solution is to constrain the upper and lower bounds of the variance in the optimization process. Future work could derive these bounds through appropriate approximations and assumptions, and design reinforcement learning or evolutionary strategies to optimize the variance.

## Resource availability

### Lead contact

Requests for further information and resources should be directed to and will be fulfilled by the lead contact, Yi Zeng (yi.zeng@ia.ac.cn).

### Materials availability

This study did not generate new unique reagents.

### Data and code availability

• This paper analyzes existing, publicly available data. These accession numbers for the datasets are listed in the key resources table.
• Code are available at https://github.com/BrainCog-X/Brain-Cog/tree/main/examples/Snn_safety/RandHet-SNN.
• Any additional information required to reanalyze the data reported in this paper is available from the lead contact upon request.

## Acknowledgments

This study was supported by the 10.13039/501100001809National Natural Science Foundation of China (grant no. 62406325), Beijing Natural Science Foundation (grant no.4252052).

## Author contributions

J.W. and D.Z. designed the study under the supervision of Y.Z. and Q.Z. J.H. implemented the models, performed the experiments and created the figures. J.H. and D.Z. wrote the manuscript and analyzed the data. X.H. and C.D. helped set up the experimental environment. Y.Z. was the project administration and funded the project.

## Declaration of interests

The authors declare no competing interests.

## STAR★Methods

### Key resources table

**Table:** 

REAGENT or RESOURCE	SOURCE	IDENTIFIER
**Software and algorithms**		

Regularized Adversarial Training(RAT)(Ding et al.^16^)	Github	https://github.com/putshua/SNN-RAT
Sparsity Regularization(SR)(Liu et al.^20^)	Github	https://github.com/putshua/gradient_reg_defense
Adversarial attacks	Github	https://github.com/Harry24k/adversarial-attacks-pytorch
Random Heterogeneous Spiking Neural Network(RandHet-SNN)	This paper	https://github.com/BrainCog-X/Brain-Cog/tree/main/examples/Snn_safety/RandHet-SNN

**Other**		

CIFAR-10 and CIFAR-100	Krizhevsky et al.^28^	https://www.cs.toronto.edu/∼kriz/cifar.html
TinyImagenet	Deng et al.^34^	https://www.image-net.org/

### Method details

#### Adversarial perturbations

Adversarial perturbations are specifically crafted modifications to input data, designed to maximize a neural network’s misclassification rate while staying within a predefined perturbation bound. Given a model C, a loss function L, an input x, and a corresponding label y, adversarial perturbations can be obtained by solving the optimization problem in Equation 1:

(Equation 1) $$\delta =\underset{{\left\|\delta \right\|}_{p}\leq \varepsilon }{\mathrm{argmax}}L\left(C\left(x+\delta \right),y\right)$$

In white-box attack scenarios, where the attacker has full access to the network’s parameters and gradients, this optimization problem can be solved to generate an adversarial example $\tilde{x}=x+\delta$. A common approach is the Fast Gradient Sign Method (FGSM), which uses the sign of the network’s gradient to compute a single-step perturbation, optimizing δ as follows^37^:

(Equation 2) $$\tilde{x}=x+\varepsilon \cdot \text{sign}\left(\nabla_{x}L\left(C\left(x\right),y\right)\right)$$

The Projected Gradient Descent (PGD) method extends FGSM by performing multiple iterative steps to generate stronger perturbations, making it an effective first-order optimization method for solving Equation 1^38^:

(Equation 3) $${\tilde{x}}_{k+1}=\prod_{\varepsilon }\left({\tilde{x}}_{k}+\varepsilon \cdot \text{sign}\left(\nabla_{x}L\left(C\left({\tilde{x}}_{k}\right),y\right)\right)\right)$$

Here, k denotes the iteration step, and the projection operator $\prod_{\varepsilon }$ ensures that each adversarial example remains within the ε-neighborhood around x.

For SNNs, adversarial attacks use the surrogate gradient method to approximate gradients of the spiking activation function. Using Backpropagation Through Time (BPTT), attackers can compute the gradients needed to create adversarial perturbations in SNNs.^16^^,^^39^

#### Spiking neuron model

SNNs commonly employ the LIF model as a nonlinear activation function due to its closer alignment with biological neuron dynamics, which we also adopt in this study. The behavior of the LIF model is governed by the Equation 4:

(Equation 4) $$\begin{array}{ll} V_{l}\left(t\right) & =\tau V_{l}\left(t-1\right)⊙\left(1-s_{l}\left(t-1\right)\right)+W_{l}s_{l-1}\left(t\right)\\ s_{l}\left(t\right) & =H\left(V_{l}\left(t\right)-V_{th}\right) \end{array}$$

where $V_{l}\left(t\right)$ and $s_{l}\left(t\right)$ represent the membrane potential and spike firing state of neurons in the l-th layer at time t, respectively. $W_{l}$ denotes the weight matrix in the l-th layer, and $V_{th}$ is the spike threshold for neuronal firing. H is the Heaviside function, indicating that neurons fire (i.e., output an activation value of 1) when their membrane potential exceeds the threshold. The parameter τ is the membrane time constant.

#### Random heterogeneous LIF model

In RandHet-SNN, each neuron within every network layer is assigned an independent random variable as its time constant. The membrane potential dynamics of LIF neurons with randomized time constants are described in the Equation 5:

(Equation 5) $$\begin{array}{ll} V_{l}\left(t\right) & =\tau_{l}⊙V_{l}\left(t-1\right)⊙\left(1-s_{l}\left(t-1\right)\right)+\left(1-\tau_{l}\right)⊙W_{l}s_{l-1}\left(t\right)\\ s_{l}\left(t\right) & =H\left(V_{l}\left(t\right)-V_{th}\right) \end{array}$$

In this equation, $V_{l}\left(t\right)$ and $s_{l}\left(t\right)$ represent the membrane potential and spike firing state of neurons in the l-th layer at time t, respectively. $W_{l}$ denotes the weight matrix in the l-th layer, and $V_{th}$ is the spike threshold for neuronal firing. H is the Heaviside function, indicating that neurons fire (i.e., output an activation value of 1) when their membrane potential exceeds the threshold. $\tau_{l}=\left(\tau_{l}^{1},\tau_{l}^{2},\ldots ,\tau_{l}^{N_{l}}\right)$ represents the vector of time constant in l-th layer, $\tau_{l}^{i}$ means the time constant of the i-th neruon and $N_{l}$ is the the total number of spiking neurons in the l-th layer.

RandHet-SNN independently sampling the time constants at each time step as shown in Equation 6, while RandHet-${\text{SNN}}^{*}$ samples the time constants at the beginning of each forward pass, maintaining them as constant for all subsequent time steps as shown in Equation 7:

(Equation 6) $$\tau_{l}^{i}\left(t\right)=\phi \left(\vartheta_{t}\right),\vartheta_{t}∼N\left(0,\sigma^{2}\right)$$

(Equation 7) $$\tau_{l}^{i}=\phi \left(\vartheta \right),\vartheta ∼N\left(0,\sigma^{2}\right)$$

where ϕ represents the sigmoid function, constraining the value of τ between 0 and 1. The variable ϑ (or $\vartheta_{t}$) is a Gaussian random variable, with $\sigma^{2}$ denoting its variance.

#### Defensive mechanisms

During the construction of adversarial attacks, RandHet-SNN samples random variables to generate a specific set of network parameters, represented by model $C_{1}$. The attacker uses the gradients of $C_{1}$ to create adversarial examples. However, when these adversarial examples are input into RandHet-SNN, the random variables are resampled, resulting in a new network instantiation, denoted as model $C_{2}$. Consequently, the adversarial perturbations crafted based on $C_{1}$ may not remain effective against $C_{2}$.

Let (X,Y) denote the data distribution of (x,y), and let $\delta_{1}$ represent the adversarial perturbations for models $C_{1}$:

(Equation 8) $$\delta_{1}=\underset{{\left\|\delta \right\|}_{p}\leq \varepsilon }{\mathrm{argmax}}L\left(C_{1}\left(x+\delta \right),y\right)$$

The transferability of adversarial example $x+\delta_{1}$ between source model $C_{1}$ and target model $C_{2}$ can be defined as follows:

(Equation 9) $$T_{r}\left(C_{1},C_{2},x\right)=\mathrm{Pr}\left[C_{1}\left(x\right)=C_{2}\left(x\right)=y\wedge C_{1}\left(x+\delta_{1}\right)\neq y\wedge C_{2}\left(x+\delta_{1}\right)\neq y\right]\text{.}$$

The above definition indicates that when both models $C_{1}$ and $C_{2}$ correctly classify clean samples but the adversarial samples successfully mislead both, the adversarial examples are considered successfully transferred.

Previous work has established an upper bound for this transferability,^36^ which we restate as Theorem 1. Theorem 1 Assume both model $C_{1}$ and $C_{2}$ are β-smooth and their gradient magnitudes are bounded by G. The adversarial perturbation ${\left\|\delta \right\|}_{2}\leq \varepsilon$. When ε is small enough, there is $L_{\min}-\varepsilon G\left(1+\sqrt{\frac{1+\bar{S}}{2}}\right)-\beta \varepsilon^{2}>0$, then the transferability can be upper bounded by

(Equation 10) $$T_{r}\left(C_{1},C_{2},x\right)\leq \frac{\xi_{1}+\xi_{2}}{L_{\min}-\varepsilon G\left(1+\sqrt{\frac{1+\bar{S}}{2}}\right)-\beta \varepsilon^{2}}\text{.}$$

where $L_{\min}={\text{min}}_{\begin{array}{l} x\in X,y^{\prime }\in Y:\\ \left(x,y\right)\in \left(X,Y\right),y^{\prime }\neq y \end{array}}\left(L\left(C_{1}\left(x\right),y^{\prime }\right),L\left(C_{2}\left(x\right),y^{\prime }\right)\right)$, $\xi_{1}$ and $\xi_{2}$ are the empirical risks of model $C_{1}$ and $C_{2}$. $\bar{S}=\sup_{x\in X,y\in Y}\;\cos\left\langle \nabla_{x}L\left(C_{1}\left(x\right),y\right),\nabla_{x}L\left(C_{2}\left(x\right),y\right)\right\rangle$ is the upper loss gradient cosine similarity between model $C_{1}$ and $C_{2}$.

Theorem 1 suggests that gradient cosine similarity is a key factor in limiting transferability. Lower cosine similarity implies poorer transferability of the attack, indicating a stronger defensive capability of the stochastic mechanism. We provide a detailed analysis of the gradient cosine similarity of RandHet-SNN with different random variances, as well as their influence on model performance in section gradient cosine similarity analysis.

#### Gradients approximation

To compute network gradients using BPTT, we employ a surrogate gradient method to approximate the gradients of the spiking activation function, as defined in Equation 11:

(Equation 11) $$H^{\prime }\left(x\right)=\frac{\alpha }{2\left(1+{\left(\frac{\pi }{2}\alpha x\right)}^{2}\right)}$$

Here, α is a hyperparameter that modulates the shape of the function. For the purposes of this study, we set α=2.

#### Black-box adversarial training

To train RandHet-SNN, we employ black-box attacks for adversarial training, a technique that promotes improved convergence of the network while mitigating the loss in clean accuracy^8^:

(Equation 12) $$\underset{W}{\mathrm{argmin}}E_{x,y∼X,Y}\left[L\left(C_{2}\left(x+\delta_{1}\right),y\right)\right],\text{where}\;\delta_{1}=\underset{{\left\|\delta \right\|}_{p}\leq \varepsilon }{\mathrm{argmax}}L\left(C_{1}\left(x+\delta \right),y\right)$$

In this formulation, $C_{1}$ and $C_{2}$ represent model instances obtained from two separate samplings of the time constants, while W denotes the other trainable parameters within the network. Black-box adversarial training is achieved by resampling the model’s random variables after generating adversarial training samples, thus enhancing robustness through stochastic parameter variations.

### Quantification and statistical analysis

#### Distribution histogram

We visualized the distributions of both time constants τ and cosine similarity using Python’s histogram plotting methods. The specific definition of the data are detailed in the legends of Figure 2 (time constants) and Figure 5 (cosine similarity).

#### Cosine similarity of gradient and adversarial perturbation

Figures 5A and 5B presents histograms of gradient cosine similarity values computed across all test samples. Specifically, we: (1) Fed each input sample through RandHet-SNN twice, (2) Computed the gradients for both forward passes, (3) Measured the cosine similarity between each gradient pair.

Figures 5C and 5D displays histograms of cosine similarity values between PGD adversarial perturbations across all test samples. Specifically, we: (1) Fed each input sample through RandHet-SNN twice, (2) Computed the adversarial perturbations for both forward passes by PGD, (3) Measured the cosine similarity between each adversarial perturbations.

The cosine similarity between two vectors a and b is calculated as:

(Equation 13) $$\text{cosine}\_\text{similarity}\left(a,b\right)=\frac{a\cdot b}{\left\|a\right\|\cdot \left\|b\right\|}=\frac{\sum_{i=1}^{n}a_{i}b_{i}}{\sqrt{\sum_{i=1}^{n}a_{i}^{2}}\cdot \sqrt{\sum_{i=1}^{n}b_{i}^{2}}}$$

#### Average gradient cosine similarity

In Table 6, Average gradient cosine similarity(AGCS) represents the mean cosine similarity of gradients across all test samples.

#### Robust accuracy

Robust accuracy measures the model’s classification accuracy on adversarial examples generated by applying adversarial attack(e.g., PGD) to the test set. All accuracy values reported in tables are expressed in percentage units (%).

#### Minimum robust accuracy

Minimum robust accuracy(MRA) quantifies a model’s worst-case performance by reporting the lowest robust accuracy observed across multiple adversarial attack strategies.
